# Performance of claims-based algorithms for identifying incident thyroid cancer in commercial health plan enrollees receiving antidiabetic drug therapies

**DOI:** 10.1186/s12913-017-2259-3

**Published:** 2017-05-05

**Authors:** Donnie Funch, Douglas Ross, Betsey M. Gardstein, Heather S. Norman, Lauren A. Sanders, Atheline Major-Pedersen, Helge Gydesen, David D. Dore

**Affiliations:** 1 Optum Epidemiology, 1325 Boylston St., 10th floor, Boston, MA 02215 USA; 2 Massachusetts General Hospital, Boston, MA 02114 USA; 3 Harvard Medical School, Boston, MA 02115 USA; 4 Global Safety, Novo Nordisk A/S, Copenhagen, Denmark; 5 Epidemiology, Novo Nordisk A/S, Copenhagen, Denmark; 6 Brown University, Providence, RI 02912 USA

**Keywords:** Methods, Thyroid cancer, Validation, Algorithm

## Abstract

**Background:**

Thyroid cancer incidence is increasing in the United States (US) and many other countries. The objective of this study was to develop and evaluate algorithms using administrative medical claims data for identification of incident thyroid cancer.

**Methods:**

This effort was part of a prospective cohort study of adults initiating therapy on antidiabetic drugs and used administrative data from a large commercial health insurer in the US. Patients had at least 6 months of continuous enrollment prior to initiation during 2009–2013, with follow-up through March, 2014 or until disenrollment. Potential incident thyroid cancers were identified using International Classification of Diseases, 9^th^ Revision (ICD-9) diagnosis code 193 (malignant neoplasm of the thyroid gland). Medical records were adjudicated by a thyroid cancer specialist. Several clinical variables (e.g., hospitalization, treatments) were considered as predictors of case status. Positive predictive values (PPVs) and 95% confidence intervals (CIs) were calculated to evaluate the performance of two primary algorithms.

**Results:**

Charts were requested for 170 patients, 150 (88%) were received and 141 (80%) had sufficient information to adjudicate. Of the 141 potential cases identified using ≥1 ICD-9 diagnosis code 193, 72 were confirmed as incident thyroid cancer (PPV of 51% (95% CI 43–60%)). Adding the requirement for thyroid surgery increased the PPV to 68% (95% CI 58-77%); including the presence of other therapies (chemotherapy, radio-iodine therapy) had no impact. When cases were required to have thyroid surgery during follow-up and ≥2 ICD-9 193 codes within 90 days of this surgery, the PPV was 91% (95% CI 81-96%); 62 (82%) of the true cases were identified and 63 (91%) of the non-cases were removed from consideration by the algorithm as potential cases.

**Conclusions:**

These findings suggest a significant degree of misclassification results from relying only on ICD-9 diagnosis codes to detect thyroid cancer. An administrative claims-based algorithm was developed that performed well to identify true incident thyroid cancer cases.

## Background

The incidence of thyroid cancer (TC) is increasing in numerous countries, including the United States (US) [1, 2]. Large administrative healthcare claims databases have been extremely valuable for the efficient and accurate examination of many health outcomes, including cancers. They can be used by providers, policy-makers, and researchers to monitor clinical activities, to increase our understanding of the risk factors associated with cancers, and to assess trends in occurrence. The key variable is the patient diagnosis, most commonly recorded using the International Classification of Diseases (ICD) 9^th^ or 10^th^ Revisions. However, relying on only a diagnosis code for case identification may lead to outcome misclassification. The first observed claim with a cancer-specific diagnosis code may not represent an incident cancer, and use of diagnosis codes alone may lead to false positive results [3]. Algorithms that accurately identify cancer outcomes have been developed for a number of cancer types by combining multiple variables available in claims data (e.g., risk factors, diagnosis/procedure codes, timing patterns) [3–5]. Outcome confirmation through adjudication (medical record review) allows the algorithm’s performance to be evaluated using positive predictive values (PPV). Algorithms with low PPVs lead to false positive results.

The primary objective of this study was to develop an algorithm for identifying true incident cases of TC using clinical input on TC diagnosis and treatment working with a TC specialist (DR) and chronological listings of all claims for individual patients for a specified period of time (claims profiles). Medical record data obtained during 4 rounds of medical record abstraction were used to evaluate algorithm components. A secondary objective was to determine and describe the proportion of microcarcinomas (tumors < 1 cm) captured among the true incident thyroid cases when applying the developed algorithms. The proportion of TC cases that fall into this category has been increasing for some time [6, 7], and these small tumors may represent a more benign form of the disease [8, 9]. It is important to understand whether microcarcinomas are identified using TC algorithms, and what proportion of the algorithm-identified cases these microcarcinomas represent, since information on tumor size is not available in claims data.

## Methods

### Data source and study population

Data for this analysis were obtained during a prospective safety study investigating the incidence of TC associated with antidiabetic (AD) drug use among AD drug initiators. The cohort was sourced from the Optum Research Database (ORD), a national commercial health insurance claims data environment containing eligibility data and pharmacy and medical claims data, with linkages to medical records for a specific subset. We identified all initiators (≥18 years of age) of metformin, sulfonylureas, pioglitazone, dipeptidyl peptidase-4 inhibitors, and glucagon-like peptide-1 receptor agonists from February 1, 2010 - December 31, 2013. All patients were required to have at least 6 months of continuous health plan enrollment with medical and pharmacy benefits in the baseline (look-back) period preceding drug initiation (cohort entry). Patients with baseline claims for dispensings of the same drug, or another drug within the class that qualified them for cohort entry, were excluded from the analysis. Also excluded were patients with baseline claims with a TC International Classification of Diseases, 9^th^ Revision diagnosis code (ICD-9 193).

Approval from the New England Institutional Review Board (NEIRB) was obtained for the use of de-identified insurance claims data, as was a Waiver of Patient Authorization from the NEIRB Privacy Board for access to protected health information and medical record data.

### Preliminary case identification

Patients were followed for incident TC from study entry (date of initiation of an AD drug) through March 31, 2014 or until disenrollment from the health plan. AD drug initiation was used to start study participation since the underlying study was designed to assess associations between AD drugs and thyroid cancer. Chart abstractions were completed annually for potential cases identified by the presence of at least one ICD-9 diagnosis code 193 during available follow-up during the previous year, with no claim for ICD-9 193 in the baseline period. Following the second round of abstraction, a claim for personal history of TC (ICD-9 diagnosis code V10.87) in the baseline period was an additional exclusion criterion. For the first 3 rounds of abstractions, a 6-month baseline period was used to exclude potential prevalent cases, while round 4 utilized all available claims data prior to cohort entry to evaluate the impact of a longer look-back period.

### Algorithm development and claims profile review

Initial variables for refinement of the TC algorithm were identified through discussions with a TC specialist (DR), and review of claims profiles for potential TC patients. Data from 3 months prior to, and up through 6 months following, the initial TC diagnosis claim were reviewed. Claims profile review eliminated patients with only a single claim for TC that was associated only with labs, as these patients had no indication of newly emergent TC. Variables reflective of risk factors or patterns of medical care related to TC diagnosis or treatment were defined (Table 1). The algorithm components are not mutually exclusive and included:

**Table 1:** Diagnostic, drug, and procedure codes used to identify thyroid cancer algorithm components

Algorithm Variables	Code Type	Code
Goiters/Nodules Diagnosis	ICD-9 Diagnosis	226, 237.4, 240.x, 241.x, 242.xx, 246.1, 246.2
Levothyroxine	HICL	002849
Thyroid surgery (partial or total thyroidectomy)	CPT-4	60200, 60210, 60212, 60220, 60225, 60240, 60242 60245, 60246, 60252, 60254, 60260, 60261, 60270, 60271
	ICD-9 Procedure	06, 06.2 ,06.3x, 06.4, 06.5x, 06.6
Chemotherapy	CPT-4	96400, 96408, 96410, 96412, 96414, 96420, 96422, 96423, 96425, 96440, 96445, 96450, 96500, 96501, 96504, 96505, 96508, 96509, 96510, 96511, 96512, 96524, 96526, 96535, 96538, 96540, 96542, 96545, 96549
	HCPCS	C8953, G9021, G9025, S5020, S9329
Radio-iodine therapy	HCPCS	A9517, A9525, A9530, A9545, Q0105, Q0106, Q0107, Q9945, Q9946, Q9948, Q9951, Q9958, Q9959, Q9960, Q9961, Q9962, Q9963, Q9964
	HICL	000747, 000748, 009223, 025482, 036581
Radiation therapy	HCPCS	X7945, G0173, G0174, G0178, G0179, S8049
	CPT-4	76950, 76965, 77261, 77262, 77263, 77280, 77285, 77290, 77295, 77299, 77300, 77301, 77305, 77310, 77315, 77321, 77326, 77327, 77328, 77331, 77332, 77333, 77334, 77336, 77338, 77370, 77371, 77372, 77373, 77380, 77381, 77399, 77401, 77402, 77403, 77404, 77406, 77407, 77408, 77409, 77411, 77412, 77413, 77414, 77416, 77417, 77418, 77419, 77420, 77421, 77422, 77423, 77425, 77427, 77430, 77431, 77432, 77435, 77470, 77499, 77520, 77522, 77523, 77525, 77750, 77761, 77762, 77763, 77776, 77777, 77778, 77781, 77782, 77783, 77784, 77785, 77786, 77787, 77789, 77790, 77799, 79000, 79001, 79005, 79020, 79030, 79035, 79100, 79101, 79200, 79300, 79400, 79403, 79420, 79440, 79445, 79999
	ICD-9 Procedure	92.2, 92.20, 92.21, 92.22, 92.23, 92.24, 92.25, 92.26, 92.27, 92.28, 92.29, 92.3, 92.30, 92.31, 92.32, 92.33, 92.39, 92.4, 92.41

*CPT-4* Current Procedural Terminology, 4th Edition, *HCPCS* Healthcare Common Procedure Coding System, *HICL* Hierarchical Ingredient Code List, *ICD-9*, International Classification of Diseases, 9^th^ Revision

Baseline:

- Claim for thyroid goiter/nodule
- No dispensings of levothyroxine

Follow-up:

- Any claim with ICD-9 193 (includes claims for inpatient/outpatient visits, screenings, laboratory tests, procedures)
- Any inpatient or outpatient visit claim with ICD-9 193
- Primary inpatient claim with ICD-9 193
- No claims with diagnosis of benign thyroid nodule ≤60 days after a claim with ICD-9 193
- ≥1 claim for thyroid surgery (partial or total thyroidectomy)
- ≥1 claim for any non-surgical treatment for TC (e.g., chemotherapy, radio-iodine therapy, radiation therapy)
- ≥1 claim any TC treatment (non-surgical or surgical)

Since most patients with TC undergo thyroid surgery and many require lifelong supplementation with the thyroid hormone levothyroxine [10], an algorithm in which patients with dispensings for levothyroxine during the baseline period were excluded was evaluated as a way of dropping prevalent TC cases. Combinations and timing of the above algorithm components were also considered, and one was retained (i.e., ≥2 ICD-9 193 codes within 90 days after thyroid surgery).

### Outcome adjudication

During each round, medical records were requested for all potentially incident claims-identified TC cases among the population of patients eligible for medical record review. For each medical record retrieved, trained medical record abstractors completed a standardized abstraction form and removed personal information from the record. The adjudicator (DR), a trained TC specialist, was given copies of each abstracted, de-identified record and the corresponding patient-specific claims profile. The adjudicator reviewed all available materials to classify each patient as: incident case, prevalent case, non-case, or insufficient information to determine case status. Tumor histology and tumor size were noted for confirmed cases, if available.

### Algorithm development

Patients for whom medical records were retrieved and determined to contain sufficient information for adjudication were eligible for inclusion in this analysis. Results from the first three rounds of charts were used to calculate performance metrics for each algorithm component. Based on these results, refined algorithms were then applied to all four rounds of data (combined) and performance metrics recalculated. The number of microcarcinomas identified was also assessed.

### Data analysis

The PPV was calculated by dividing the number of confirmed cases by the number of claims-identified potential cases that met an algorithm’s case definition. The PPVs and exact 95% confidence intervals (CIs) were calculated to evaluate the performance of the algorithms and their individual components. We also calculated a proxy for sensitivity, defined as the percentage of confirmed cases identified by the algorithm (or algorithm component), and calculated a proxy for specificity, defined as the proportion of non-cases who were identified by the absence of an individual characteristic or combination of characteristics. Combinations of algorithm components were selected to maximize both sensitivity and specificity. Because thyroid cancer is more prevalent among females [1], the possibility that the diagnostic and treatment process might vary by gender was considered in assessing algorithm performance. Patients classified as “history of TC” during adjudication were reclassified as non-cases.

## Results

**Fig. 1:**
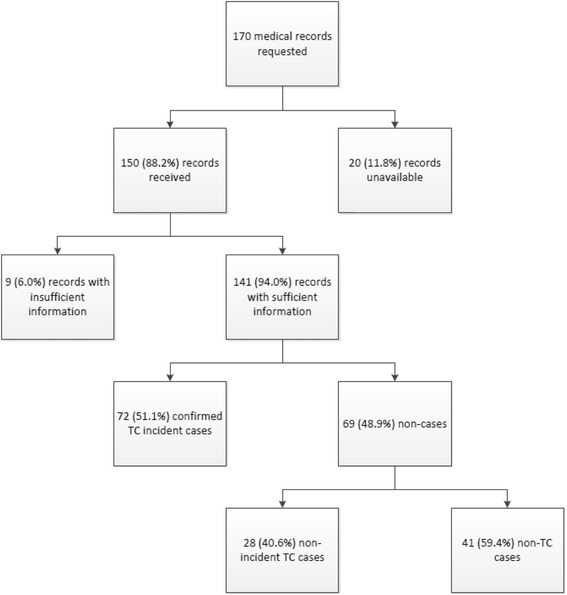
Identification process and outcome for thyroid cancer case adjudication

Among 340,484 study drug initiators, there were 796 patients with at least one claim in the follow-up period containing ICD-9 diagnosis code 193. Among them, 585 were identified as potential prevalent cases of TC and were excluded for having the same code in the baseline period. Claims profile review was completed on 211 patients, 41 of whom were dropped because their ICD-9 193 codes were associated only with labs or because they had a pattern of care indicating a history of TC prior to cohort entry. Medical records were requested for 170 individuals. The results of the process through adjudication are displayed in Fig. 1. Of the requested records, 88.2% were obtained, of which 141 (94.0%) had sufficient information for adjudication by the TC specialist. Of the 141 potential cases reviewed, 102 (72.3%; 95% CI 64.4-79.1%) were female, 92 (65.2%; 95% CI 56.7-72.0%) were between the ages of 40–59, and 89 (63.1%; 95% CI 54.6-71.0%) had a baseline diagnosis of thyroid goiters/nodules or other benign thyroid conditions.

Overall, 72 (51.1%) incident TC cases were positively adjudicated; pathology reports were available for 60/72 (83.3%). Of the 69 “non-cases”, 28 (40.6%) were classified as “non-incident TC cases”, and 41 (59.4%) were determined to be “non-TC cases”. The age-gender distribution was similar between cases and non-cases (data not shown), and therefore neither age nor gender was incorporated in any of the algorithms.

Within the population adjudicated during the first 3 rounds of medical record review, 25% of adjudicated cases were identified as having TC prior to cohort entry, despite having no claim for TC in the 6-month baseline period. In contrast, the fourth round of review included patients for whom *all* available claims data prior to cohort entry were used to exclude prevalent cases. Only 9% of adjudicated cases from that round were identified as having TC prior to cohort entry.

The algorithm components, as well as the algorithms themselves, are presented in Table 2 along with corresponding performance metrics. Since all individuals included in this analysis had ≥1 claim for ICD-9 code 193, the PPV represents the confirmation rate for that single component (PPV: 51%, 95% CI: 43-60%, *n* = 141). Adding the requirement that the diagnostic code be associated with an inpatient or office visit increased the PPV to 57% (95% CI 48-66%, *n* = 119), but reduced both sensitivity and specificity. Restricting the ICD-9 code to an inpatient visit with the TC code in the first position decreased the number of false positives but had no impact on the PPV and decreased the number of cases. Sensitivity dropped to 19% and the sample size decreased to *n* = 21.

**Table 2:** Algorithm characteristics for the identification of thyroid cancer. Optum Research Database, February 1, 2010 - December 31, 2012

		Adjudicated Status							
Algorithm components identified from claims	Total	Cases	Non-Cases	Positive Predictive Value (PPV)	95% CI	Sensitivity^a^	95% CI	Specificity^b^	95% CI
a) ≥ 1 claim with ICD-9 193	141	72	69	51%	43-60	100%	94-100	0%	0-7
b) Any inpatient or outpatient claim with ICD-9 193	119	68	51	57%	48-66	94%	86-98	26%	17-38
c) Primary inpatient claim with ICD-9 193	21	12	9	57%	34-77	17%	09-28	87%	76-93
d) Claim for goiter in baseline	49	30	19	61%	46-74	42%	30-54	72%	60-82
e) No dispensings for levothyroxine in baseline	104	62	42	60%	50-69	86%	75-93	39%	28-52
f) No claims with diagnosis of benign thyroid nodule ≤ 60 days after ICD-9 193	123	69	54	56%	47-65	96%	87-99	22%	13-34
g) Claims for thyroid surgery in follow-up	103	70	33	68%	58-77	97%	89-100	52%	40-64
h) Claims for any non-surgical treatment (chemo, radio-iodine, radiation)	40	38	2	95%	82-99	53%	41-65	97%	89-99
i) Any treatment (non-surgical or thyroid surgery)	104	71	33	68%	58-77	99%	91-100	52%	40-64
Algorithms									
1) Thyroid surgery and no baseline levothyroxine	90	60	30	67%	56-72	83%	72-93	57%	44-68
2) Thyroid surgery and ≥2 ICD-9 193 codes ≤ 90 days after surgery	68	62	6	91%	81-96	86%	75-93	91%	81-96

*CI* Confidence interval, *ICD-9* International Classification of Disease, 9th Revision

^a^This proxy for sensitivity represents proportion of adjudication-confirmed cases who have the characteristic

^b^This proxy for specificity represents proportion of adjudication confirmed non-cases who do not have the characteristic

The presence of an ICD-9 diagnosis code indicating thyroid goiter or nodule, known risk factors for TC [11, 12], had a PPV of 61% (95% CI 42-72%, *n* = 49). The absence of levothyroxine at baseline had a PPV of 60% (95% CI 50-69%, *n* = 104) and performed reasonably well in identifying cases (sensitivity 86%), but performed poorly in eliminating non-cases (specificity 57%).

Requiring the presence of a procedure code for thyroid surgery increased the PPV to 68% (95% CI 58-76%, *n* = 103) and substantially increased the sensitivity to 97%, but still classified almost half of the non-cases as cases (specificity 52%). A claim for non-surgical treatment for TC had a high PPV (95%) but sensitivity was poor, with only 53% of the cases identified. Including other forms of therapy for TC with or without thyroid surgery (*n* = 104) had no impact on the PPV of the algorithm based on thyroid surgery alone.

The algorithm requiring at least 2 claims with the ICD-9 code 193 within 90 days following thyroid surgery produced the highest PPV (91%; 95% CI 81-96%,n = 68) and acceptable sensitivity (86%) and specificity (91%). This algorithm performed similarly for males (PPV: 95%; 95% CI 74-100%, n = 21) and females (PPV: 91%; 95% CI 78-97%, n = 46).

Tumor size was available in 68 (94%) of the medical records. Among the positively adjudicated cases with known size, 30 (44.1%; 95% CI 32.3-56.6%) were microcarcinomas and all were histologically classified as papillary. Both algorithms under consideration captured at least 80% of the microcarcinomas; the best performing algorithm (≥2 ICD-9 193 codes following thyroid surgery) identified 25 (83%) of the 30 patients with microcarcinomas as cases.

## Discussion

Within a safety study of diabetic therapies, we examined claims and medical record data among adults with an incident claims diagnosis of TC. We identified an algorithm comprised of a combination of claims characteristics with a PPV of 91%, which is a substantial increase over the PPV of 51% observed for an algorithm that required only the presence of at least one TC diagnostic code. The optimal algorithm, based on highest PPV, was a combination of the presence of a thyroid surgery procedure code and at least 2 ICD-9 diagnosis codes for TC within 90 days of the procedure. This combination identified the vast majority of the confirmed cases and dropped from consideration the majority of patients with either prior TC or other unrelated thyroid problems.

The optimal algorithm was developed by combining clinical expertise and review of healthcare claims data. The identification of TC is generally associated with thyroid surgery, suggesting this a critical component of any algorithm for this outcome [1, 13]. In our study, 97% of the confirmed cases had thyroid surgery. However individuals with goiters or nodules, known risk factors for TC, are also more likely to have thyroid surgery due to continued growth or patient discomfort, regardless of malignancy status [14]. Adding the requirement that patients have at least 2 diagnosis codes for TC around the time of the thyroid surgery eliminates many individuals with benign growths. Because this algorithm was developed using a single database with an insured population, its performance may vary in populations with different characteristics and coding practices.

By extending the length of the baseline period from 6 months to all available data, we demonstrated that the longer look-back period may be preferable for screening out prevalent cancers, reducing the number of false positive incident TC cases.

There are limitations to this study to be considered. While the medical record retrieval and abstraction rate was high, a number of potential cases were excluded from this analysis because medical record data could not be obtained. If these records were unavailable for adjudication purely due to administrative reasons, we would expect the PPV estimates to be less precise, but unbiased. If they were not (e.g., if medical records were withheld because of ongoing litigation related to cancer), then our estimates may be vulnerable to bias in either direction. This is also true for those potential cases deemed to have insufficient information for adjudication. And, because this analysis was restricted to patients with an ICD-9 code for TC, we have not included potential cases where TC was not recorded in their claims due to coding errors or other coding practices. Only ICD-9 codes were in use in the ORD database at the time these data were collected. The ICD-10 diagnostic code for TC is also a single code (C73) and should be used in addition to or in place of the ICD-9 code when applicable.

Algorithms are typically developed and applied in claims or other electronic health record databases where access to large amounts of electronic medical data is available. Often these are insured commercial or public health plan populations that do not represent all individuals in the general population. This is an inherent limitation of the type of data available, rather than the algorithm itself. Study findings applying those algorithms to insured populations need to take this into account. For example, studies assessing incidence of thyroid cancer in insured populations may overestimate rates for histologic types that are often benign in nature such as papillary microcarcinoma, where detection bias may result from the more frequent opportunity for surveillance [15, 16]. This overestimation is even more likely in insured populations where the patients are all receiving AD therapy and have regular visits.

Despite these limitations, the population of TC patients identified was similar to what one would expect with regard to age and gender. Thyroid cancer was more common among women and tended to be diagnosed most often in patients between the ages of 45–64, similar to the patterns identified by SEER for the time period of the study [1]. The proportion of microcarcinomas was also within the range expected [17]. And comparisons of thyroid cancer rates between groups drawn from the same insured population should be unbiased.

We examined the usefulness of the algorithms for detecting microcarcinomas. While the final algorithm (≥2 codes following thyroid surgery) identified most of the confirmed microcarcinomas, it could only capture microcarcinomas that were clinically identified and treated with thyroid surgery or those that were identified “incidentally” when thyroid surgery was performed for another reason. Data from 1998–2010 noted that almost 75% of identified microcarcinomas underwent thyroidectomies [18]. Practices are changing. The current American Thyroid Association guidelines recommend performing fine needle aspiration biopsies only for nodules ≥ 1cm [19]. Smaller nodules are followed for future changes in size or other characteristics. This “watchful waiting” approach may result in fewer biopsies and surgeries for microcarcinomas; only 50% of the microcarcinomas identified in this study were noted pre-operatively and were the reason the patient had thyroid surgery. Since the remainder were incidental, it is likely that applying this algorithm will continue to identify a TC population that includes a considerable proportion of microcarcinomas.

While we provided sensitivity, specificity and PPV as measures of performance, these were estimates based on the number of adjudicated cases and the limitations of these measures must be considered. Measures of true sensitivity and specificity would require the examination of records for patients who did not meet the claims coding criteria as well as for those who did. Multivariate statistical approaches such as stepwise logistic regression or decision tree classification programs (e.g., CART) may also be used to improve the performance of claims-based algorithms.

## Conclusions

This study describes the properties of 2 algorithms for incident TC case identification, and confirms a high level of agreement between administrative healthcare claims and medical records for one algorithm, suggesting claims data may be useful in assessing trends in occurrence of this growing healthcare problem. An additional recommendation is to use an expanded baseline period, taking advantage of all available information prior to application of the algorithm to increase the detection of false positives due to prior history of the outcome.

## Abbreviations

AD: Antidiabetic
CART: Classification and Regression Trees
CI: Confidence interval
ICD-10: International Classification of Diseases, 10^th^ Revision
ICD-9: International Classification of Diseases, 9^th^ Revision
NEIRB: New England Institutional Review Board
ORD: Optum Research Database
PPV: Positive predictive value
TC: Thyroid cancer
US: United States
